# Systemic Lupus Erythematosus and Systemic Autoimmune Connective Tissue Disorders behind Recurrent Diastolic Heart Failure

**DOI:** 10.1155/2012/831434

**Published:** 2011-09-21

**Authors:** Luis Miguel Blasco Mata, Olga Acha Salazar, Carmen Rosa González-Fernández, Francisco Novo Robledo, Enrique Pérez-Llantada Amunárriz

**Affiliations:** Unidad de Alta Resolución Hospitalaría (UARH), Pab. 13, -1, Hospital Marqués de Valdecilla, Avenida Marqués de Valdecilla, 39008 Santander, Spain

## Abstract

Diastolic heart failure (DHF) remains unexplained in some patients with recurrent admissions after full investigation. A study was directed for screening SLE and systemic autoimmune connective tissue disorders in recurrent unexplained DHF patients admitted at a short-stay and intermediate care unit. It was found that systemic autoimmune conditions explained 11% from all of cases. Therapy also prevented new readmissions. Autoimmunity should be investigated in DHF.

## 1. Introduction

Diastolic heart failure (DHF) is a clinical syndrome in which patients have symptoms and signs of heart failure (HF), normal left ventricular ejection fraction (LVEF), and evidence of diastolic dysfunction. Recurrent DHF episodes are rare when recognized triggers are corrected and therapy is appropriate. However, it remains unexplained in some patients with recurrent admissions after full investigation. There are no systematic data available upon recurrent DFH, and few reports provide an underlying diagnosis. Interestingly, Schwagten described DFH as a first presentation of mixed connective tissue disease [1].

Inflammation and autoimmunity are currently receiving attention as mechanisms for DHF [2–6]. Impaired myocardial dysfunction [7–11] has been confirmed in Systemic Lupus Erythematosus (SLE). Nevertheless, autoimmunity is not routinely tested in DHF patients. There are not previous reports in the literature which investigate autoimmunity as the underlying cause of DHF.

## 2. Objectives

The main purpose of the study attempts to identify SLE or systemic connective tissue disorders (SCTD) in recurrent unexplained DHF patients. The secondary point was length of readmission-free time under appropriate therapy.

## 3. Patients and Methods

### 3.1. Definition of Case and Study Population

Case was defined as unexplained recurrent DHF whom fulfilled SLE or STCD classification criteria.

### 3.2. Definition of Unexplained Recurrent DHF

DHF was defined as (1) symptoms and signs of heart failure described in the guidelines published by the American Heart Association [12] and European Society of Cardiology [13]; (2) LVEF over 55% and altered diastolic filling pattern; and (3) absence of hypokinesis or ventricle dilation, on echocardiogram.

Recurrent was considered two or more admissions within last year under appropriate treatment and absence of precipitant factors. Patients had received diuretics and angiotensin converter enzyme inhibitors. Hydric balance, salt intake, blood pressure, atrial fibrillation, or valve involvement were not triggers and must be stable at admission.

Unexplained implied unidentifiable cause after investigation, including routine blood count, complete serum and urine biochemistry, troponin and thyroid hormones, chest X-rays, continuous electrocardiography monitorization and new echocardiography. Infection, coronary ischemia, anemia, hypothyroid or hyperthyroid state were ruled out. Pulmonary embolism was investigated, even in anticoagulated patients, by angioCT scan when D-dimmer was positive or there was suggestive echocardiography.

### 3.3. Diagnosis of Autoimmune Condition

Common SLE/SCTD symptoms and signs were evaluated. STCD comprised APS, Sjögren Syndrome (SS), Mixed Connective Tissue Disease (MCTD), and Undifferentiated Connective Tissue Disease (UCTD). Physicians were guided by a nonvalidated internal consensus questionnaire, which contained 17 items (see the appendix). Clinical pattern was accepted as probable SLE/SCTD when fulfilled 3 or more items.

Immunological tests were only requested when this clinical pattern was presented and accepted after two repeated positive determinations. Immunology tests included anti-nuclear antibodies (ANAs), anticardiolipin antibodies (aCL), anti-*β*2-glycoprotein I antibodies (anti-*β*(2) GPI), anti-DNA antibodies, thyroglobulin autoantibodies (anti-TGB) and thyroid peroxidase autoantibodies (anti-TPO). Anti-TGB and anti-TPO were decided due to given association between rheumatic heart disease and autoimmune thyroid disease [14].

Recurrent unexplained DFH patients whom fulfilled probable SLE/SCTD with positive immunology were revaluated by a trained physician on systemic autoimmune connective tissue disorders. Moreover, this physician only selected those cases whom fulfilled correct diagnosis and evident SLE/APS/SCTD classification criteria [15, 16]. Alarcon-Segovia [17] and European Consensus Group [18] classification criteria were used for MCTD and SS, respectively. UCTD was restrictively considered, for research purposes, when clinical and immunological features did not fulfilled classification criteria for those conditions but usual clinical practice actually suggested disease and recommended further followup expecting new criteria in the outcome or investigations. Patients with similar profile but insufficient data were not included, although they will be briefly commented.

### 3.4. Study, Protocol, and Phases

**Figure 1:**
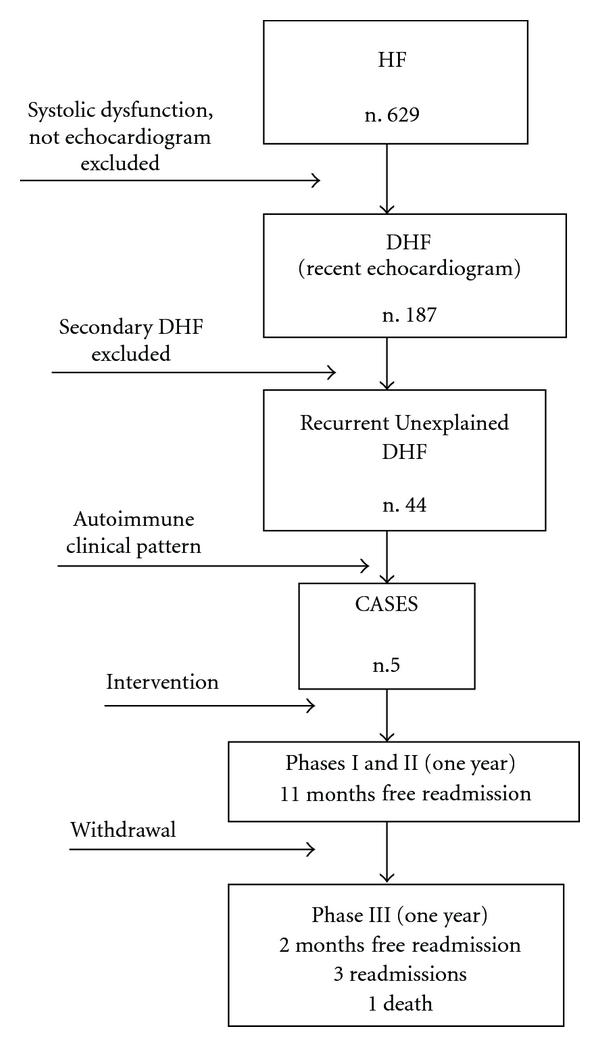
Protocol and phases. HF: Heart failure, DHF: Diastolic heart failure.

A prospective observational study was performed between July 2007 and November 2009 at a short-stay and intermediate care unit (UARH) of the University Hospital Santander, Spain. The study was divided in Phase I, Phase II, and Phase III (Figure 1), using an identical evaluation protocol.

Phase I was conducted between July 2007 and September 2008, for main objective. Cases were recruited from consecutive admitted heart failure patients. Systolic dysfunction and absence of recent echocardiography were exclusion criteria. History, clinical findings, laboratory results, and imaging were integrated by the clinicians in charge, which finally made the diagnosis of unexplained recurrent DHF. Second screening was subsequently directed for SLE/SCTD in those DHF patients, who underwent questionnaire and regular followup with immunology tests. Those who fulfill probable SLE/SCTD criteria were revaluated by an expert physician, who finally considered cases when patients with correct diagnosis met classification criteria for SLE/APS/SCTD.

Phase II was conducted until October 2008, for secondary objectives. Cases were treated and outcome was observed. Detailed regimens will be discussed in results. Free-admission time, readmission rate, and therapeutic changes by other specialists were registered.

Phase III was conducted between October 2008 and November 2009. Cases were referred to respective competent departments (Cardiology, Hematology, Rheumatology, and Internal Medicine) for specialized long-term followup. We observed outcome and registered therapy and diagnosis changes. Free-readmission and readmission rate time were also listed again.

## 4. Results

### 4.1. Phase I

629 heart failure patients were admitted in Phase I. Only 187 patients had prior echocardiogram and showed nonsystolic heart failure (30%); 442 patients were excluded. From them, 44 patients were classified as recurrent and unexplained DHF (Figure 1).

Finally, 5 patients (11%) fulfilled the definition of case (Table 1).

### 4.2. Cases Description

#### 4.2.1. Case 1

Case 1 was an 80-year-old woman with past history of penicillin allergy, secondary hypertension, polyarticular rheumatism in adolescence, thyroid surgery in adulthood, and current mild renal failure, with noninvestigated hematuria. She had been diagnosed osteoarthritis because of joint pains, joint effusion on knees, and ANA 1/160, by rheumatology department ten years ago. She had been followed due to rheumatic double mitral lesion by cardiology department for 13 years, but she had not needed interventions and recent LVEF was normal. She referred Raynaud phenomena, chronic asthenia, malar rash and photosensitivity, which have been improved with age; she had livedo reticularis, joints effusions on wrist, knees and ankles. She had shown chronic leucopenia (<4000/mL) on past blood counts. Immunology tests demonstrated myeloperoxidase-anti-neutrophil-cytoplasmic antibodies (MPO-ANCA) > 100, and anti-*β*(2)GPI 24. She fulfilled diagnosis criteria and 5 classification criteria for SLE, with antiphospholipid antibodies. MPO-ANCA may be observed until 25% of SLE patients, with activity correlation. Renal, heart, and thyroid disease were not invasively assessed searching more criteria.

#### 4.2.2. Case 2

Case 2 was a 67-year-old woman, with past history of metamizol allergy, late onset hypertension, and asthma. She had been diagnosed with amiodarone-induced hyperthyroidism, although she had showed repeated high titers of anti-TPO. Amiodarone was only given for some months and definitively discontinued. She had started being followed due to rheumatic double mitral lesion by cardiology department last year after one episode of heart failure admission. She had undergone anticoagulant therapy. She had rashes, photosensitivity, chronic symmetric distal polyarticular joint pain and swelling, Raynaud phenomena, asthenia, and cognitive impairment. She presented livedo reticularis and joint effusions on physical examination, and erythema was confirmed in followup. Brain CT scan demonstrated multiple lacunar infarcts, although acenocoumarol levels were adequate. Immunology tests showed ANA > 1/1280 but aCL and repeated anti-TPO were negative. Libmann-Sacks endocarditis was not found on repeated echocardiography. She fulfilled diagnosis criteria and 4 classification criteria for SLE.

#### 4.2.3. Case 3

Case 3 was an 80-year-old woman, with past history of metamizol allergy, constrictive pericarditis, and heart failure. She referred facial rashes, significant photosensitivity, Raynaud phenomena, sicca syndrome, relapsing asthenia periods, and swelling and joint pains on wrists, knees, and ankles. Dermatological and joint signs were confirmed on examination. Blood account showed leucopenia. Initial immunology tests demonstrated ANA 1/320 and anti-*β*(2)GPI 33, which remained repeatedly positive. Echocardiogram showed a 70 mm left auricle and diastolic dysfunction. Case-resembled SLE or SS, but she was classified as UCTD with antiphospholipid autoantibodies, due to absence of sufficient criteria.

#### 4.2.4. Case 4

Case 4 was a 67-year-old woman, with intricate past history. She suffered one miscarriage at second pregnancy trimester and an antenatal death due to preterm fetus. Then, she developed an episode of 9-month-length unexplained fever and joints pain when she was 42 years old. She had suffered two episodes of labeled transient ischemic attack, when she was 51 and 54 years old. However, the second one consisted of new onset headache and sensitivity loss during 3 months. Finally, she was diagnosed with recurrent periorbital rash and hypothyroidism by Allergy Department in last year. She referred recurrent rash flairs, eye dryness, and Raynaud phenomena. She had residual periorbital erythema, a round bright hyperkeratosic pink plaque on ankle, and livedo reticularis. Immunology tests showed ANA 1/1280 and anti-TPO 507. The only echocardiographic hallmark was a 48 mm left auricle and diastolic dysfunction. Case resembled SLE/ APS/SS, but she was classified as UCTD, in the absence of sufficient criteria.

#### 4.2.5. Case 5

Case 5 was an 80-year-old woman with rheumatic double mitral lesion which required prosthetic replacement when she was 68 years old. However, she underwent new replacement due to unexplained early valve dysfunction two years later. She had been admitted after second surgery in multiple occasions because of heart failure, although repeated echocardiography showed both normal valve and LVEF. She had been also evaluated due to polyarthritis, uveitis, anemia, and ANA 1/320 five years ago, but without definitive conclusions. ANA was repeated and remained positive (1/320). She was classified as UCTD.

### 4.3. Phase II

All of cases have been treated on angiotensin enzyme converter inhibitors, acenocoumarol, and diuretics, which were continued.

Azathioprine 50 mg pd. was given to Case 1. Initial methylprednisolone bolus (1.5 g), azathioprine 50 mg pd., and hydroxychloroquine 200 mg pd. were given to Case 2. Hydroxychloroquine 200 mg pd. was selected for the other three cases. Diuretics were reduced in all of cases. Case 2 required increasing diuretics after complete discontinuation of treatment by Surgery Department, 6 months later. Moreover, methimazole was discontinued in Case 2 but thyroid hormones continued normally during Phase II. Echocardiograms were not repeated again at Phase II.

There were not new admissions due to DHF within Phases I and II. The admission-free time was, respectively, 11, 26, 11, 5, and 5 months. Case 1 was readmitted because of pulmonary embolism two months after diagnosis, and she was reclassified as SLE and secondary APS.

### 4.4. Phase III

We refer all of cases to Cardiology, Rheumatology, and Internal Medicine Departments. SLE and UCTD diagnosis were criticized but APS were accepted. Patients did not received an alternative diagnosis, and we understand that they were considered false-positive autoantibody cases. Immunosuppressive agents were discontinued. Cases 1, 2, and 3 readmitted. The admission-free mean time was two months after therapy changes. Cases 1 and 2 needed Intensive Care Unit in the first readmission. Case 1 died due to heart failure and retroperitoneal hemorrhage after a long three-month admission. Case 2 was readmitted again 7 months later.

## 5. Discussion

Systemic autoimmune connective tissue disorders explained 11% from recurrent unexplained DFH admissions in this study.

DHF is determined by abnormal diastolic filling of left ventricle. It may be precipitated by hemodynamic stress from cardiovascular (e.g., tachycardia, hypertension) or other origin (e.g., infection, anemia). DHF usually is easily managed when triggers are corrected; therefore, recurrences are not expected. Nevertheless, absence of evident cause may be followed by new episodes. We usually observe unexplained recurrent DHF episodes in patients whom accumulate suggestive data of autoimmune background. We believe that chronic uncontrolled systemic inflammation hinders adequate diastolic filling as other studies reported [10, 19]. However, few studies attempt to investigate underlying autoimmunity in heart failure patients. 

The case profile was an elderly woman with past history of allergies, rashes, chronic polyarticular involvement, thyroid disease, secondary hypertension, rheumatic mitral lesion, and ANA positive. Surprisingly, no case had presented rheumatic fever features in childhood. SLE and APS cause similar valve involvement [20]. On the other hand, Hashimoto's thyroiditis has been already linked to rheumatic heart disease [14], so we could speculate about possible unrecognized systemic autoimmune diseases. Thus, we believe rheumatic description on echocardiography should be taken cautiously and should be further investigated.

In the same way, we think that elderly age of cases demonstrated delayed diagnosis and evolution of uncontrolled inflammation. Although young patients may display more autoimmune activity and diastolic dysfunction, overt heart failure is unlikely to expect, as in nonautoimmune patients. We did not found DHF patients under 50 years old. Therefore, elder patients should not be excluded without an appropriate history. With respect of this, osteoarthritis or hypertension must be dated in order to decide whether they are primary or consequence of previous inflammation.

Moreover, specific therapy seemed to prevent readmission and discontinuation of medications. Inversely, withdrawal was dramatically associated to readmissions after long symptom- free periods.

Therefore, we could state that autoimmunity, particularly SLE/SCTD, should be always considered within DHF patients.

Study was designed to answer whether autoimmune connective tissue disorders explained recurrent episodes, but not for every episode or other autoimmune diseases. Perhaps, affected males or vasculitis should be investigated following different protocols. We cannot conclude that autoimmune DFH patients fulfill the proposed profile. In fact, we found one patient with bronchiolitis and vasculitis, who did not satisfy our criteria. We did not know if DHF or even any type of heart failure should be always investigated. We noticed *a posteriori* that study excluded 6 similar patients. As well as, 8 excluded patients showed rheumatic heart disease.

Likewise, it does not answer if immunology tests are valid or/and cost-efficient although clinical pattern was absent. Prevalence studies for underlying autoimmune diseases are needed. On other hand, other DHF causes should be investigated.

Finally, the study design did not include controls to compare admission-free mean times. Conclusions on therapy remain intuitive.

We tried to explain the events on Phase III. Phase III intended congruency among physicians and second opinion for patients. Unfortunately, we did not expect the dramatic outcome of Cases 1 and 2. We searched reasons on the classification criteria misuse by inexpert and strict physicians. Patients were informed about possible changes but they complained after discontinuation of a regimen felt as beneficial. Ethical issues and consensus should be improved on new experimental clinical research about autoimmunity.

## 6. Conclusions

Autoimmunity is an important mechanism of diastolic dysfunction. Autoimmune connective tissue disorders should be considered in patients with recurrent DHF. Specific therapy may improve patients and prevent readmissions. Elderly patients should not be excluded from investigations. Rheumatic heart disease should be reconsidered in suspicion of systemic disease. More studies are warranted for clarifying prevalence of underlying autoimmunity, for systematic exposition of other DHF causes, and for cost-effectiveness of immunological tests.

##  Conflict of Interests

The authors declared that there is no conflict of interests.

## Figures and Tables

**Table 1:** Cases characteristics (*n* = 5).

Age	74 years (65 y–80 y)
Gender	100% female (5)
Ethnicity	White South European
Allergies or urticaria	80% (4)
Polyarticular rheumatism	100% (5)
Thyroid problems	60% (3)
Miscarriages and obstetric morbidity	20% (1)
Raynaud	40% (2)
Livedo reticularis	60% (3)
Leucopenia	40% (2)
Previous positive autoantibody tests	80% (4)
Antithyroid autoantibodies positive	40% (2)
Autoimmune condition previously diagnosed	0% (0)
Secondary Hypertension	80% (4)
Rheumatic mitral lesion	40% (2)
Atrial fibrillation	100% (5)
Pulmonary thromboembolism	20% (1)
Autoantibodies positive	100% (5)
ANAs positive >1/340	80% (4)
aCL positive	40% (2)
SLE	40% (2)
APS	20% (1)
UCTD	60% (3)

## Appendix

### Questionnaire

SYMPTOMS

**Table:**

*✓*	CLINIC DATA	
1	**AGE**	
	**Age 15–45 years-old**	
	Age >45–55 years-old	
2	**VASCULAR RISK FACTORS**	
	**Not**	
	Yes	
3	**Pill adverse efects**	
	**Yes**	
	Not	
4	**DVT/PE**	
	**Yes**	
	Not	
5	**Migrain**	
	**Yes**	
	Not	
6	**Cognitive impairment**	
	**Yes**	
	Not	
7 (no.)	**Miscarriages**	
	**Yes**	
	Not	
8	**Obstetric complications**	
	**Yes**	
	Not	
9	**Familiar background (SLE, etc.)**	
	**Yes**	
	Not	
10	**Dermatology **(erythema, photosensitivity, aphtae, alopecia, Raynaud)	
	**Yes**	
	Not	
11	**Joint involvement**	
	**Yes**	
	No	

PHYSICAL EXAMINATION

**Table:**

*✓*	SIGNS	
12	**LÍVEDO RETICULARIS**	
	**Yes**	
	Not	
13	**FACIAL ERYTHEMA **	
	**Yes**	
	Not	
14	**MOUTH ULCERS**	
	**Yes**	
	Not	
15	**ALOPECIA**	
	**Yes**	
	Not	
16	**PERIUNGEAL LESIONS**	
	**Yes**	
	Not	
17	**ARTHRITIS HANDS/FEET**	
	**Yes**	
	Not	

REQUEST AUTOANTIBODIES

**Table:**

**APS (*)**	**APA, ANA**
SLE	ANA, C3,C4,Ch50
Vasculitis	ANA, ANCA p y c
ANA +	anti-DNA, Anti Ro, anti La
**APA +**	Repeat 6 weeks

(*) APS: when at least 2 items (no. 1–9, 12)
